# A retrospective review of influences on clinicians to order whole body CT scans in trauma and its effectiveness in this regard

**DOI:** 10.1186/1757-7241-21-S1-S16

**Published:** 2013-05-28

**Authors:** R Sloan

**Affiliations:** 1 Royal Oldham Hospital, Oldham, UK

## Background

Clinical examination alone in trauma has been shown to be ineffective, with equivocal or misleading findings in 20-50% of patients suffering from blunt polytrauma [1].

As a result, the use of whole-body CT (WBCT) in trauma assessment of patients is rapidly increasing. However, WBCT exposes patients to large amounts of radiation and so clinicians around the UK are under pressure to have strong justification for the use of WBCT. In Leeds General Infirmary (LGI) there is no specific protocol for ordering a WBCT currently in place. We aimed to assess which of the triage criteria influence the staff the most when ordering a CT - specifically: mechanism of injury (MOI) or injury severity based on clinical assessment and physiology profile. We aimed to evaluate which, if any of these factors where associated with clinically occult injury (COI) being diagnosed following WBCT.

## Method

In a retrospective study, data from the trauma audit and research network and LGI’s own e-records were used to assess severity of MOI (using Lerner et. al[2] to create a scale), clinical assessment severity was decided using the revised trauma score and probability of survival predicted based on this score [3]

Which of the triage criteria influenced the clinician’s decision and if a clinically occult injury (COI) was found on CT were assessed and a predictive model was then used to decide which of the triage criteria had the strongest influence in diagnosing a COI.

## Results

33 patients who underwent WBCT were included in the study. Mean age: 35, 31 (94%) were men and mean injury severity score was 27. 75% had a severe MOI, 52% had normal physiology and 55% had severe clinical assessment. 55% had a COI found. No statistically significant relationship was found between these variables and the diagnosis of COI following WBCT. Moderate or severe MOI increased probability of COI being diagnosed by 1.368 and 4.965 respectively. Moderate and severe physiology increased the probability of diagnosing a COI by 1.368 and 8.682 respectively. Moderate clinical assessment increased the probability of diagnosing a COI by 3.526 while severe clinical assessment decreased it by 69%, although results were not statistically significant.

## Conclusions

Clinical assessment is primarily used by clinicians in LGI with a heavy influence of MOI. The current system is relatively accurate but further work should be done to ensure the triage system is accurate. Clinical assessment appears to be the most powerful tool even compared to imaging, however a larger study is needed to achieve statistical significance.
